# A scoping review protocol to map the evidence on the use of action research methodology by healthcare professionals and in healthcare team settings

**DOI:** 10.12688/hrbopenres.13275.1

**Published:** 2021-07-05

**Authors:** Kinley Roberts, Mary Casey, David Coghlan, Catherine Cornall, Clare Hudson, Diarmuid Stokes, Aine Carroll

**Affiliations:** 1 National Rehabilitation Hospital, Dun Laoghaire, Dublin, A96 E2H2, Ireland; 2 School of Medicine and Medical Science, University College Dublin, Dublin, D04 C7X2, Ireland; 3 School of Nursing Midwifery & Health Systems, University College Dublin, Dublin, D04 C7X2, Ireland; 4 Trinity Business School, Trinity College Dublin, Dublin, D02 F6N2, Ireland; 5 UCD Library, University College Dublin, Dublin, D04 C7X2, Ireland

**Keywords:** Action research, methodology, healthcare settings, healthcare professionals, interdisciplinary team working, rehabilitation, scoping review, protocol

## Abstract

**Background:** Action research (AR) starts with an existing practical situation with which there is a concern or potential for improvement. It seeks transformative change through the simultaneous process of doing research and undertaking actions, both of which are linked together by a critical reflective process. It simultaneously allows one to systematically investigate a given social situation while promoting democratic change and collaborative participation. AR approaches have been used for many years in business management and education. More recently, AR has become an increasingly popular method of inquiry in healthcare, particularly in nursing, to investigate professional practice while simultaneously; introducing innovations; planning and undertaking action; and evaluating new ideas. The overall goal is to augment collaboration whilst improving the patient experience and outcomes.

**Methods:** The Arksey and O'Malley methodology framework will be used to guide this scoping review process: stage 1 will identify the research questions; the eligibility criteria and search strategy will be defined in stage 2; studies will then be selected in stage 3; data will be extracted and charted from these included studies in stage 4; stage 5 involves aggregating and summarising these results along with criteria relevant for health professionals and policy-makers. An optional consultation (stage 6) exercise may potentially be included.

**Conclusion:** This scoping review will comprehensively map the evidence on the use of AR methodology by healthcare professionals and in healthcare team settings. It is predicted that the findings will inform researchers in carrying out future AR and highlight gaps in the literature. An article reporting the results of the completed scoping review will be submitted for publication to a scientific journal and presented at relevant national and international conferences.

## Introduction

Action research (AR) is a philosophy and methodology of research that starts with an existing practical situation with which there is a concern or potential for development or improvement ( Coghlan, 2019). It is also described as a process that seeks transformative change through the simultaneous process of taking actions and doing research, which are linked together by a critical reflective process ( Coghlan, 2019). Winter and Munn Giddings defined AR as the ‘study of a social situation carried out by those involved in that situation to improve both their practice and the quality of their understanding’ ( Winter & Munn Giddings (2001), pg. 8). Baum *et al.* says AR *“seeks to understand and improve the world by changing it. At its heart is collective, self-reflective enquiry that researchers and participants undertake so they can understand and improve upon the practices in which they participate and the situation in which they find themselves”* ( Baum *et al.* (2006), pg. 854). It relies on a cycle of reflection, planning, acting, further observing and reflection, and the researchers are engaging together in these processes ( Baum, 2016).

AR is experiential and participatory in nature, which led Kemmis & McTaggart (2000) to describe it as participatory research. Other variations of AR include community-based AR, participatory AR, community-partnered participatory research, mutual inquiry, collaborative research, co-operative inquiry, action learning, and participatory learning research ( Bergold & Thomas, 2012). However, the foundations and basic concept of AR underpins them all.

The characteristics of AR contrast with positivist science and overlap more with the post-positivist and interpretivist research paradigm ( Cordeiro *et al.*, 2015; Tekin & Kotaman, 2013). Kurt Lewin, a psychologist, first coined the term in 1944, and it appears in his 1946 paper "Action Research and Minority Problems", where he describes it as *"a comparative research on the conditions and effects of various forms of social action and research leading to social action"* ( Lewin (1946), pg.35) that uses *"a spiral of steps, each of which is composed of a circle of planning, action, and fact-finding about the result of the action"* ( Lewin (1946), pg.38). This idea of research in which teachers are more active participants was highlighted much earlier, however, in the works of John Dewey ( Dewey, 1910), who emphasized the importance of experience and interaction between human beings and with their natural and artistic environment. Dewey's criticism of the separation between knowledge and action as well as his other philosophical ideas are instrumental for the creation of the AR approach ( Dewey, 1929). Many have since elaborated on Lewin’s ideas, such as Rapaport, who defined the aim of AR as contributing both to the practical concerns of people in an immediate problematic and concerning situation but also to the goals of social science by joint alliance within a mutually acceptable ethical framework ( Brown & Tandon, 1983)

AR has traditionally been used in qualitative research to facilitate change in education and business management ( Carr & Kemmis, 1986; Elliott, 1991; Shani & Coghlan 2019). More recently, its use has become more popular in nursing and nursing education ( Moch *et al.*, 2016; Munn-Giddings *et al.*, 2008; Munten *et al.*, 2010). It has been employed less in other areas of health ( Sørensen & Haugbølle, 2008; Swain & French, 2004), a surprising fact, especially as it can enable development of innovative practices and services over a wide range of health care practices and situations ( Holter & Schwartz-Barcott, 1993). The need for enhanced and effective team collaboration has been emphasized increasingly in healthcare policy both nationally and internationally ( Committee on the Future of Healthcare, 2017; Harris & Schmitt, 2004).

Effective teamwork in healthcare is associated with reduced medical errors, increased patient safety as well as improved staff outcomes such as reduced stress and job satisfaction ( Buttigieg *et al.*, 2011; Carter & West 1999; Firth-Cozens, 2001; Manser, 2009).

The National Rehabilitation Hospital (NRH) is the national tertiary centre providing specialist rehabilitation services to patients who due to accident, illness or injury have acquired a physical or cognitive disability. The NRH delivers rehabilitation through the collaborative work of interdisciplinary team (IDT) members. Team members include doctors, nurses, physiotherapists, occupational therapists, speech and language therapists, pharmacists, psychologists, medical social workers, music therapists, dieticians, and health care assistants. In physical medicine and rehabilitation (PRM), team working is considered essential and “interdisciplinary working” is the preferred pattern of team working ( Neumann *et al.*, 2010). It is considered the most effective way of delivering integrated, person-centred care and leads to improved outcomes for patients ( Xyrichis & Ream, 2008). IDT working is defined as a dynamic process involving two or more health professionals with complementary backgrounds and skills, sharing common health goals, and exercising concerted mental and physical effort in assessing planning and evaluating patient care ( Nancarrow *et al.*, 2013). IDT also offers benefits to staff in terms of morale, job satisfaction, feelings of empowerment and improved wellbeing and benefits to the organisation in cost savings, length of stay and reduced staff turnover ( Körner *et al.*, 2016). Reflective team processes can enhance team cohesiveness, professional identities, create a safe place for reflection and enhance team focus ( Heneghan *et al.*, 2014). Healthcare professionals do not instinctively know how to work together in IDTs and the “work” in teamwork must be acknowledged ( Sargeant *et al.*, 2008).

In 2020 the NRH transitioned to a new hospital facility, considered by many to be the ultimate change project ( Stichler & Ecoff, 2009). A hospital interdisciplinary working research group had been set up one year prior to the move, and we had identified that this transition had real potential for our IDT’s dynamic to be disturbed and for patient care to become fragmented. Enhancing the effectiveness of IDT working has been studied in a community-based rehabilitation setting using AR methodology with promising results ( Nancarrow *et al.*, 2012). We set out to implement a similar Interdisciplinary management tool to the NRH, aimed at enhancing IDT working, and to explore the outcome of this on patients, staff, and the organisation.

An initial scoping search of the literature revealed little regarding team training or interventions in the rehabilitation setting and during transition periods or change projects. Therefore, we felt that this was a worthwhile research study with potential benefits for patients and staff. We also predicted that this study may offer guidance to other teams transitioning in the future in Ireland e.g., the National Paediatric Hospital, Dublin and the new National Maternity Hospital, Dublin. To support our project, we chose a scoping review to map the relevant literature in this area. The objective of this scoping review is to investigate the use of AR methodologies in healthcare team and research settings. Of particular interest to us is the use of AR in rehabilitation settings, in multidisciplinary teams and in teams that are transitioning. This scoping review will be conducted in accordance with the Arskey and O’Malley framework methodology ( Arksey & O'Malley, 2005), which has been further developed by Levac *et al.* (2010), and the Joanna Briggs Institute (2015).

## Protocol

### Design

The scoping review is informed by the framework proposed by Arksey & O’Malley (2005) which has been further developed by Levac *et al.* (2010) and the Joanna Briggs Institute (2015). Hence, the review process is organised into five stages. More recent work by Peters (2017), Peters *et al.* (2020a) and Peters *et al.* (2020b) proposes a nine-step approach using a more explanatory framework with greater visibility given to extracting the evidence, analysis of evidence, presentation of results to finally summarise the evidence in relation to the purpose of the review, drawing conclusions and noting implications going forward.

- Stage 1. Identifying the research question.
- Stage 2. Identifying relevant studies.
- Stage 3. Study selection.
- Stage 4. Charting the data.
- Stage 5. Collating, summarising and reporting the results.

The Arksey and O’Malley framework proposes an optional sixth stage. This involves consulting with key stakeholders in order identify any further references and studies that they feel should be included. It also allows opportunity to gather their feedback regarding the scoping review findings. This additional consultation with content experts ensures that the search strategy includes all the appropriate terms and enhances the relevance of the research overall.

We feel that a consultation with key stakeholders is therefore a worthy and valuable exercise but, this will be decided upon later and will depend on time and budget. The Stakeholder group would include multidisciplinary team members- speech and language therapy manager, a Clinical Nurse Manager, a Senior physiotherapist, two international experts in the field of AR and an expert in the field of Integrated Care.

### Stage 1: Identifying the scoping review question and aligning the research objectives

Prior to establishing the research question, a preliminary analysis of the literature on AR methodologies in healthcare was completed to help refine the scope of this protocol. The ‘PCC’ mnemonic, where the population, concept and context are described ( Peters *et al*., 2020a) is the recommended format for cultivating the question for scoping review and this was used here. This method aids in the development of the inclusion and exclusion criteria and helps to inform the literature search strategy.

- **Population/types of participants:** healthcare professionals who work in healthcare teams and those involved in healthcare AR - nursing, medical, allied healthcare professionals, healthcare management.
- **Concept:** studies that use AR methodology to enhance teamwork, collaboration, and performance in healthcare.
- **Context:** studies using AR that take place in healthcare teams and in any healthcare settings including acute care, primary care, rehabilitation, and community settings.

Based on this initial research, the following specific research questions and objectives emerged.

***Specific research questions and objectives***

- Determine the extent to which AR has been undertaken in healthcare.
- Identify what specific health disciplines use AR e.g. medical, nursing, psychology, allied healthcare professionals etc.
- Determine in which healthcare settings AR is carried out e.g. acute care, primary care, rehabilitation, and community settings.
- Determine the extent to which AR has been undertaken in rehabilitation.
- Examine the extent to which AR has been undertaken within multidisciplinary teams or IDTs.
- Assess the overall impact of AR in healthcare and summarise/disseminate findings.
- Ascertain if AR is considered effective at enhancing teamwork in healthcare settings and if there is evidence to support that it improves patient outcomes, patient experience, staff experience and the overall success of an organization.
- Identify if research findings, conclusions and recommendations provide evidence of knowledge building and change.
- Ascertain if there are any gaps in research and make recommendations for future research.
- Establish the value of carrying out a full systematic review on this subject.
- Determine the barriers and facilitators to the effective undertaking of AR.

***Inclusion and exclusion criteria.*** This scoping review will consider both qualitative and quantitative primary research in the English language and between the years 2000 and 2021 that illustrate the AR methodology. Unpublished (grey literature) will also be included. It was agreed to exclude conference abstracts and non-English articles. See Table 1 for full inclusion and exclusion criteria with justifications.

**Table 1.** Inclusion and exclusion criteria for study selection.

Criterion	Inclusion	Exclusion	Justification
**Language**	English	Any other language	Reviewers are only English speaking
**Focus**	Articles that discuss or use action research methodology in any healthcare team and any healthcare setting and aimed at improving teamwork, collaboration, staff experience, patient outcomes or patient experience.	Articles outside he healthcare context	
**Time period**	2000–2021	Outside this time period	Action research has recently become more prominent within healthcare. Initial scoping search showed that most of the literature falls within this period.
**Types of** **articles**	1. Peer reviewed journal articles/reviews 2. Unpublished (grey literature) including theses, dissertations, editorials, and book chapters.	1. Conference abstracts. 2. Editorials or other opinion pieces.	Scoping reviews aim to capture more than peer reviewed and published literature in order to expansively explore a broad research question.
**Geographic** **location**	Any location	None	Action research has application globally

### Stage 2: Identifying relevant studies

***Search strategy.*** A three-step search strategy will be utilised in this scoping review. An initial search of two databases, PubMed and CINAHL, will be undertaken, followed by an analysis of the text words contained in the title and abstract of retrieved papers, and of the index terms used to describe the articles. Using the PCC framework (population, concept, context), ideas will be expanded on using search terms and appropriate thesaurus terms. Synonyms for each of the concepts will also be included

A second search using all identified keywords and index terms will then be undertaken across all included databases. Databases to be searched include Cochrane Database of Systematic Reviews, PUBMED, CINAHL, Scopus, Web of Science, Social Sciences, ERIC, PsycINFO, Health Source: Nursing/Academic Edition.

Thirdly, the reference lists of identified reports and articles should be searched for additional sources. Reviewers intend to contact authors of primary articles or reviews for further information if this is relevant. A search for grey material might also be necessary.

 Finally, a complete search strategy for at least one major database will be included as an appendix to the protocol. An expert university librarian will be part of the research team and aid in designing and refining the search strategy from the start. This will be peer-reviewed by another librarian as recommended ( McGowan *et al*., 2016). Only reports and articles in the English language (due to time and budget constraints) and between the years 2000 and 2021 will be included.

 The input of a research librarian is crucial to ensure the entire search strategy and results are transparent and auditable. They will provide guidance on the most suitable Medical Subject Headings (MeSH) terms for the search as well as advising on how best to adapt these terms for individuals databases. Sample search terms for the PubMed database are outlined in Table 2.

**Table 2.** Sample search terms for the PubMed database.

PCC concept	Initial keywords
**Population/participants - healthcare** **professionals who work in health care** **(either independently or within teams)**	Healthcare worker* OR Nurse* OR Doctor* OR Physician* OR Consultant* OR medic* OR “advanced practitioner” OR Physiotherapist* OR Physical Therapist* OR Speech therapist* OR Speech Language therapist* OR Occupational therapist* OR Psychologist* OR Allied Healthcare Professional* OR social worker* OR Medical Social Worker* OR Worker, Social* OR Health Services Manager* OR Healthcare Assistant* OR Dietician* OR General Practitioner* OR GP* OR Health Personnel* OR Pharmacist* OR "Midwife" OR "health professional" OR "allied health professional" OR Allied Health Occupations* OR "health* manage*" OR "multidisciplinary team" OR "Interdisciplinary team"
**Concept - studies using action research** **methodologies in healthcare contexts**	Action research* OR participatory research* OR participatory action research* OR mutual inquiry* OR collaborative research* OR co-operative inquiry* OR “Cooperative Inquiry” OR participatory learning research* OR Community-Based Participatory Research* OR Action learning* OR action science* OR action evaluation* OROR appreciative inquiry* OR clinical inquiry* OR collaborative inquiry* OR collaborative developmental action inquiry* OR interactive research* OR intervention research* OR learning history* OR Cooperative behaviour* OR “Organisation Development” OR “Organization Development” OR “Organizational development” OR “Organisational Development” OR “Co-Design”
**Context - any healthcare team in any** **healthcare setting (primary, acute** **hospital, tertiary) to improve teamwork**	Teamwork* OR collaboration* OR team performance* OR problem solving* OR health education* OR interprofessional practice* OR interdisciplinary working* OR multidisciplinary working* OR Social Change* OR Interprofessional Relations* OR Interdisciplinary Communication* OR hospital team* OR “Acute Care” OR primary care team* OR “Primary Health Care” OR rehabilitation team* or community team* OR “Community medicine” OR “Long-term Care” OR Long term health care OR “Mental Health Services” OR Psychiatric OR “Nursing Homes” OR “Healthcare” OR “health care” OR “health-care” OR “health and care” OR “hospital” OR “health facility” or “acute care” OR “health system” OR “Tertiary care” OR “secondary care” OR “primary care” OR “general practice” OR “aged care” OR “residential care” OR “social care” OR “clinic” or “community care” OR “disability service” OR “mental health” OR “older persons service” OR “rehabilitation” OR “palliative care” OR “Clinical Nursing” OR “Clinical Medicine”

### Stage 3: Source of evidence selection

Study selection will begin with screening of titles and abstracts by two reviewers, independently, using the pre-specified inclusion and exclusion criteria. Where consensus is not reached between the two reviewers regarding inclusion, this will be solved by the decision of a third reviewer. Also, if one reviewer cannot decide whether a study is eligible for inclusion, this will be further discussed between the two reviewers until agreement is reached or it is reconciled by the third reviewer.

The screening process will be carried out using Rayyan (an online open access screening software tool) and will remove non-relevant studies. Articles will be retrieved from each database and imported into the reference management software Endnote, where duplicates will be removed. A rationale will also be provided for all exclusion criteria ( Tricco *et al*., 2018).

Pilot testing will be conducted prior to embarking on source selection across a team. This will include:

- Random sample of 25 titles/abstracts will be selected.
- The whole team will screen these using the eligibility criteria.
- Team will meet to discuss any discrepancies and make modifications to the eligibility criteria.
- Team will only start screening when 75% (or greater) agreement is achieved.

The process of study selection will be reported using the Preferred Reporting Items for Systematic reviews and Meta-Analysis extension for Scoping Reviews (PRISMA-ScR) ( Tricco *et al*., 2018), which will then be updated once the review is completed

### Stage 4: Data extraction/ “charting”

A data extraction framework or charting table will be developed within Microsoft Excel software (Version 2105) as suggested by Joanna Briggs Institute (JBI) ( Peters, 2017). This will include 15 categories used to assess the full review articles retrieved from the literature fulfilling the eligibility criteria for inclusion ( Table 1).

1. Author(s) and year of publication
2. Study title
3. Origin/country of origin (where the source was published or conducted)
4. Aims/purpose
5. Population and sample size within the source of evidence (if applicable)
6. Study setting/healthcare context
7. Methodology/methods
8. AR cited principles
9. Number of AR cycles
10. Intervention type and details of these (e.g. duration of the intervention) (if applicable)
11. Outcomes and details of these (e.g. how measured) (if applicable)
12. Knowledge building
13. Social change
14. Key findings that relate to the scoping review question/s.
15. Citing of authors

Two team members will pilot test the charting table using a sample (10%) of the complete list of retrieved studies to be included. This will guarantee that the coding framework is consistently applied. Modifications to the categories and revisions to the charting table may occur later if necessary. Any queries or disagreements arising from this pilot test will be discussed and resolved by the whole team consultation. These measures will ensure that we are transparent and clear in our methods regarding what and how we will extract data.

### Stage 5: Collating, summarising, and reporting the results

The collected data will then be analysed using the data extraction framework. This will result in information on a body of research where AR methodologies have been used in healthcare teams and in healthcare settings. The clinical effectiveness of AR in this context will be highlighted as well as under-researched areas or those warranting further investigation

Presentation of the results will be in a visual and aggregate form (e.g., using charts and tables) as well as a descriptive format aligning to the objectives and scope of this review.

The results summary will clearly lay out the purpose or aims of, and the methodologies used by each of the reviewed sources. Results will be categorised into principal conceptual groups that will be established during the results extraction. A clear explanation will be provided for each category or group.

## Assessment of methodological quality

As it is a scoping review, no assessment is required.

## Study status

The final protocol was registered prospectively with the Open Science Framework on the 7 ^th^ January 2021 ( https://osf.io/wt5n7). Initial searches of databases had commenced and author roles had been delegated at the time of this article publication.

## Discussion

Systematic review articles concerning AR in education, business, and nursing exist but appear to be lacking in other areas of healthcare. In particular, there seems to be a paucity of evidence synthesis related to AR to improve IDT working either in rehabilitation settings or teams in transition.

This systematic scoping review will focus on use of AR methodologies in all healthcare teams and in healthcare settings. It will include all studies published between the years 2000 and 2021. We envisage that the results of this scoping review will contribute to literature but also aid policy guidelines for maintaining and enhancing IDT working particularly for teams in transition periods or change projects. Findings of this scoping review will be disseminated electronically, in print, and through peer presentation, conferences, and congresses.

## Data availability

No data are associated with this article.
