# Supplementary material for: A scoping review protocol to map the evidence on the use of action research methodology by healthcare professionals and in healthcare team settings
Source: HRB Open Res. 2021 Jul 5;4:68. [Version 1] doi: 10.12688/hrbopenres.13275.1 (PMC11116937; doi:10.12688/hrbopenres.13275.1)
Supplement: Peer review history

# Reviewer report: Reviewer response for version 1

**DOI:** 10.21956/hrbopenres.14448.r39984

**Published:** 2024-05-23

**Authors:** Nadeeka Rathnayake

**Affiliations:** 1 Rajarata University of Sri Lanka, Mihintale, North Central Province, Sri Lanka

Thank you so much for the opportunity to review this manuscript. The authors have planned an important study that may contribute to filling gaps in the existing knowledge base in the application of AR.

 Overall, the manuscript is well-written and a few of my concerns to further improve its quality are given below;

- In the introduction, please mention how your review would benefit the global community, not only Ireland.
- Please summarize your information about the National Rehabilitation Hospital (NRH) because some details might not be necessary. 
- You have mentioned "We set out to implement a similar Interdisciplinary management tool to the NRH, aimed at enhancing IDT working, and to explore the outcome of this on patients, staff, and the organization", please clarify whether this scoping review is a part of a project aiming to do this? otherwise, you suddenly start talking about NRH which is a bit misleading.
- Please don't repeat your search strategy both in the Introduction and the Design. 
- Please expand your Discussion to describe any possible challenges, limitations, and strengths of your design.  

Thank you.

Is the study design appropriate for the research question?

Yes

Is the rationale for, and objectives of, the study clearly described?

Yes

Are sufficient details of the methods provided to allow replication by others?

Yes

Are the datasets clearly presented in a useable and accessible format?

Not applicable

Reviewer Expertise:

Health Promotion, Action Research, Violence

I confirm that I have read this submission and believe that I have an appropriate level of expertise to confirm that it is of an acceptable scientific standard.

# Reviewer report: Reviewer response for version 1

**DOI:** 10.21956/hrbopenres.14448.r29771

**Published:** 2021-07-09

**Authors:** Graham Williamson

**Affiliations:** 1 School of Nursing and Midwifery, University of Plymouth, Plymouth, UK

This is well-written and well constructed and should be indexed. I take the point that as a scoping review assessment of methodological quality is not required but I would just urge some caution with that assertion: in recent SRs I have found some of what purports to be research, even in apparently decent journals, is so poor as to be not worth the name. Think about whether you can usefully use the JBI tools for critical appraisal purposes, and GRADE to help you assess the recommendations. This would add an additional layer of rigour.

Is the study design appropriate for the research question?

Yes

Is the rationale for, and objectives of, the study clearly described?

Yes

Are sufficient details of the methods provided to allow replication by others?

Yes

Are the datasets clearly presented in a useable and accessible format?

Yes

Reviewer Expertise:

action research, systematic review

I confirm that I have read this submission and believe that I have an appropriate level of expertise to confirm that it is of an acceptable scientific standard.
